# Low-Grade Thermal Energy Harvesting and Self-Powered Sensing Based on Thermogalvanic Hydrogels

**DOI:** 10.3390/mi14010155

**Published:** 2023-01-07

**Authors:** Jiedong Zhang, Chenhui Bai, Zhaosu Wang, Xiao Liu, Xiangyu Li, Xiaojing Cui

**Affiliations:** 1 Qiushi College, Taiyuan University of Technology, Taiyuan 030024, China; 2 College of Information and Computer, Taiyuan University of Technology, Taiyuan 030024, China; 3 Shanxi Transport Information Communication Company Limited, Taiyuan 030006, China; 4 College of Civil Engineering, Taiyuan University of Technology, Taiyuan 030024, China; 5 College of Physics and Information Engineering, Shanxi Normal University, Taiyuan 030031, China

**Keywords:** self-powered, hydrogels, thermogalvanic, low-grade thermal energy, sensors

## Abstract

Thermoelectric cells (TEC) directly convert heat into electricity via the Seebeck effect. Known as one TEC, thermogalvanic hydrogels are promising for harvesting low-grade thermal energy for sustainable energy production. In recent years, research on thermogalvanic hydrogels has increased dramatically due to their capacity to continuously convert heat into electricity with or without consuming the material. Until recently, the commercial viability of thermogalvanic hydrogels was limited by their low power output and the difficulty of packaging. In this review, we summarize the advances in electrode materials, redox pairs, polymer network integration approaches, and applications of thermogalvanic hydrogels. Then, we highlight the key challenges, that is, low-cost preparation, high thermoelectric power, long-time stable operation of thermogalvanic hydrogels, and broader applications in heat harvesting and thermoelectric sensing.

## 1. Introduction

The world has been meeting an ever-increasing electricity demand by burning more fossil fuels since the second Industrial Revolution [1,2,3,4,5,6]. Therefore, the accelerating consumption of non-renewable resources for energy production has led to an urgent need for increased energy production from renewable resources [7,8,9,10,11]. On the other hand, heat can be considered renewable in that it is ubiquitous and inevitable. A vast amount of low-grade heat (<200 °C) is distributed in the environment (solar-thermal and geothermal energy), produced as by-product waste heat from industrial processes and dissipated from the human epidermis and electronic equipment [1,12,13,14]. In parallel, thermoelectrics is the most straightforward technology applicable to direct heat-to-electricity energy conversion [15,16,17,18]. However, low-grade heat is rarely commercially utilized because of its intrinsic low temperature and inefficiency [19]. To this end, technologies to convert low-grade heat to electricity must be efficient, scalable, and cost-effective [20,21]. Among them, applications of inorganic semiconductive thermoelectric generators are expensive, have mechanical brittleness, and are challenging to synthesize. Their thermoelectric efficiency is limited not only by the small Seebeck coefficient (thermodynamic quantity, determined by the change in the electron chemical potential caused by temperature changes) of about 100–200 μV K^−1^ but also by the electrical and thermal conductivity [22,23,24].

Nevertheless, thermogalvanic technologies provide an approach to directly convert heat to electrical energy without complex synthesis procedures, greenhouse gas emissions, or unsatisfactory long-term reliability. Another advantage of thermogalvanics for low-grade heat harvesting is their high thermopower on the order of mV K^−1^. This simplifies device design and assembly to generate high voltages, despite a slight temperature difference [25,26,27,28,29].

**Figure 1:**
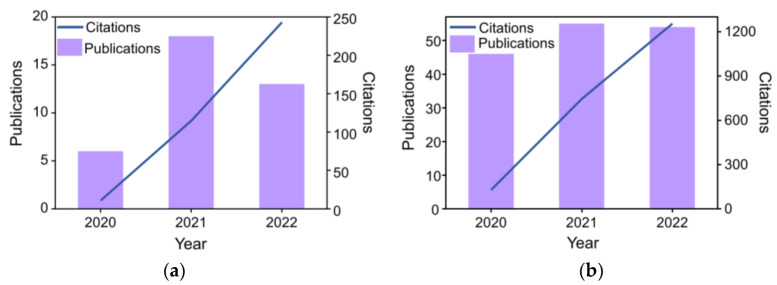
Citation frequency and publication distribution by year for hydrogel-based thermal energy harvesting (**a**) and sensing (**b**).

In recent years, significant progress has been made in liquid thermogalvanics by improving electrodes, electrolytes, and redox species [30,31,32,33,34]. In particular, studies on quasi-solid thermogalvanics have eliminated the leakage risk of liquid electrolytes by introducing physically crosslinked networks [15]. Hydrogels (a three-dimensional network structured gel that can absorb large amounts of water) are preferred for quasi-solid-state thermoelectric cells that convert heat to electricity through a temperature-dependent electrochemical process, with a conversion efficiency (ZT) determined primarily by electrical conductivity, thermal conductivity, and the Seebeck coefficient, which is hundreds of times higher than that of semiconductor flexible thermoelectrics. In addition, some flexible devices based on thermogalvanic hydrogels have been developed for human thermal energy generation, body temperature monitoring, and solar energy harvesting [16,35,36]. Inexpensive and environmentally friendly electrode and electrolyte materials form simple device structures, as well as excellent thermoelectric power performance [30,31,32]. As shown in Figure 1, the number of publications and citations using hydrogels to harvest and sense thermal energy has increased from 2000 to 2022 [18,19,20,21,22,23,24,25,26,27,28,29,30,31,32,33,34,35,36,37,38,39,40,41,42,43,44,45,46,47,48,49,50,51,52,53,54,55,56,57,58,59,60,61,62,63,64,65,66,67,68,69,70,71,72,73,74,75,76,77,78,79,80,81,82,83,84,85,86,87,88,89,90,91,92,93,94,95,96,97,98,99,100,101,102,103,104,105,106,107,108,109,110,111,112,113,114,115,116,117,118,119,120,121,122,123,124,125,126,127,128,129,130,131,132,133,134,135,136,137,138,139,140]. This has increased interest in flexible thermogalvanics for low-level heat harvesting [37,38,39]. Although all flexible thermogalvanics have the same basic configuration of two electrodes sandwiched by an electrolyte, multiple classifications exist, defined by different electrolytes, electrode materials, solvents, and redox pairs [40,41].

In this mini-review, we aim to systematically sort through the latest research on thermogalvanic hydrogels to give the reader as comprehensive an overview as possible of the multiple roles of polymer networks, redox pairs, and thermogalvanic electrode materials in hydrogel formation and to give the reader a complete picture of flexible thermogalvanic hydrogels. The content was divided into six parts: in the first part, we provided an overview of how low-grade thermal energy harvested and introduced the emerging thermogalvanic hydrogels; in the second part, we investigated ways and characterization methods to improve the efficiency of individual thermogalvanic hydrogels; in the third part, we presented the selection of polymers and encapsulation methods; in the fourth part, we exhibited the integration method of thermogalvanic hydrogels; in the fifth part, we gave an introduction to the potential applications of thermogalvanic hydrogels; and in the end, a brief summary of the research on thermogalvanic hydrogels was provided and an outlook on the development of thermogalvanic hydrogels was given.

## 2. Improving the Efficiency of Single Thermocell

### 2.1. The Basic Principle of the Thermogalvanic Effect

**Figure 2:**
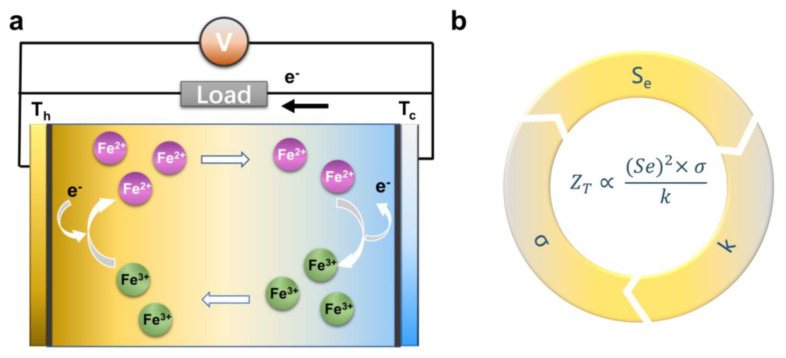
(**a**) Thermogalvanic working principle. (**b**) Influencing factors of thermoelectric figure of merit ZT.

Based on the thermogalvanic effect, quasi-solid thermal cells convert thermal energy into electrical energy through two fundamental processes: redox reactions at the electrodes and mass transfer in the electrolyte [42,43,44,45,46,47]. When a temperature gradient is applied to the entire thermogalvanic cell, the temperature dependence of the redox reaction leads to oxidation at the anode and reduction at the cathode of the redox couple. The reduced material is transported through the electrolyte by convection, diffusion, and migration to the anode, which is oxidized. Then, the eroded material is transported back to the cathode, resulting in a continuous reaction, as shown in Figure 2a. Therefore, a constant current is generated in the thermogalvanic cell. As long as there is no degradation of the cell components, this reaction can theoretically continue indefinitely [16].

### 2.2. Reaction Thermodynamics

The conversion efficiency of a thermogalvanic cell depends on three interdependent parameters: the Seebeck coefficient (*Se*), the electrical conductivity (σ), and the thermal conductivity (*k*), as shown in Figure 2b [15,16,42,43,44]:

(1) $$ZT=\frac{{\left(Se\right)}^{2}\times \sigma }{k}$$

According to thermodynamic theory, the *Se* is given by:

(2) $$Se={\left(\frac{\partial E}{\partial T}\right)}_{t=\infty }=\frac{\left[\left(S_{B}+{\hat{S}}_{B}\right)-\left(S_{A}+{\hat{S}}_{A}\right)-n{\overset{=}{S}}_{e}\right]}{nF}$$

where *n* is the number of electrons transferred, *F* is Faraday’s constant, $S_{A}$ and $S_{B}$ are the partial molar entropies of species *A* and *B*, ${\hat{S}}_{A}$ and ${\hat{S}}_{B}$ are their Eastman entropies, and$\;{\overset{=}{S}}_{e}$ is the transported entropy of the electrons in the external circuit [48,49,50,51]. Eastman transport entropy is explained by the interaction of the ion and its solvated shell layer with the solution. In most calculation schemes, the values of *A* and *B* are much smaller than $S_{A}$ and $S_{B}$ and can be ignored, and the order of magnitude of *Se* is μV K^−1^, which can also be ignored in most calculations. As such, Equation (1) can be written as:

(3) $$Se={\left(\frac{\partial E}{\partial T}\right)}_{t=\infty }=\frac{S_{B}-S_{A}}{nF}$$

Among these parameters, the change in entropy is influenced by various factors, including structural changes in the redox species, the effect of the solvent shell layer, and its interaction with the solvent [52,53,54]. Whether in aqueous or non-aqueous solvents, the positive or negative value of the entropy change is related to the difference between the absolute charges of the oxidizing and reducing substances, and the magnitude of the value reflects the strength of the main Coulomb interaction between the charged redox species and their solventized shells; if the absolute charge of the oxidizing agent is greater than that of the reducing agent, the Seebeck coefficient is positive, and vice versa. In general, redox species with a high absolute charge and complicated complex structure possess a large strength of the Coulomb interaction and thus have a high Se. For example, the Se of [Fe(ClO_4_)_3_/Fe(ClO_4_)_2_) (~1.46 mV K^−1^) is much higher than that of Fe^2+^/Fe^3+^ (~1.0 mV K^−1^) [55,56]. As a result, when increasing the Seebeck coefficient, the potential difference between cells increases, leading to a consequent increase in the current that can be generated.

Nevertheless, the maximum power output is also governed by various overpotentials within the system during cell operation [57,58,59,60]. Theoretically, the performance of a given cell is also determined by three major overpotentials within the thermogalvanic cell: ohmic overpotential, charge transfer overpotential, and mass transfer overpotential [61,62,63]. The further complexity in the thermogalvanic arises from the temperature dependence of each of these overpotentials. The thermal gradient serves as an essential factor in the performance of the cell [64,65,66]. A gel with low thermal conductivity can maintain a high-temperature difference between the electrodes, and the reduction of the temperature gradient will also reduce the thermogalvanic potential. Reaction kinetics is used to explain conductivity; the reaction resistance of redox substances at the electrode and the transport resistance in the electrolyte is related to the magnitude of conductivity [67,68,69,70].

Most of the reported thermogalvanic elements have energy conversion efficiencies (relative to the Carnot engine) of less than 1% [71,72,73]. However, some recent studies have reported record conversion efficiencies of 11% by establishing high concentration ratios at the cold and hot ends [74]. The Seebeck coefficient, conductivity, power density, and number of charge/discharge cycles of some thermogalvanic hydrogels is shown in Table 1 [21,23,45,56,75]. Despite these low efficiencies, since the primary use of thermogalvanic hydrogels is to harvest wasted energy, the efficiency requirements for commercial viability are relatively low. Some estimates suggest that 2–5% conversion is sufficient for practical energy harvesting applications [76,77].Nevertheless, this depends on the manciencies of effiufacturing and installation costs as well as the device’s lifetime.

To improve the thermal efficiency as well as the long-term stability of thermogalvanic hydrogels, there are two main target areas of research: (1) increase the potential difference and current density that may be generated in the thermogalvanic cell; (2) optimize the equipment design in cooperation with the polymer network to improve the mechanical properties, long-term operating performance, thermoelectric properties and other capabilities of the thermogalvanic hydrogel [78,79]. The potential can be increased by developing and optimizing the basic thermodynamic principle, i.e., applying a redox couple and electrolyte combination with a high Se. In addition to increasing the potential of individual thermogalvanic cells, another hot research area focuses on device integration and applications [80,81]. The output voltage of thermogalvanic-integrated devices can be increased by optimizing the polymer network or using different bridging methods. Current density can be increased by using high specific surface area electrodes with good quality. The environmental stability of the device can be improved by using suitable packaging methods. As described below, these aspects are currently being investigated as potential strategies to improve thermogalvanic element integration.

### 2.3. Thermopower Measurement and Calculation

**Figure 3:**
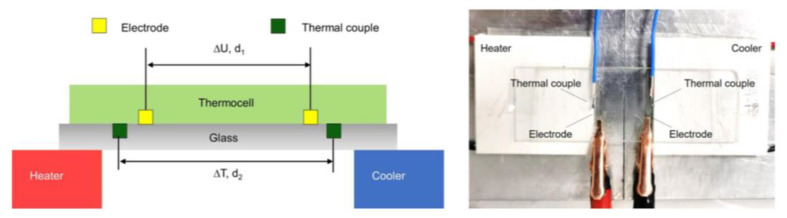
Schematic and digital images of the platform for thermopower measurements. (Reproduced with permission from [82]. Copyright 2021 Elsevier Inc.).

The measurement and calculation of Se will generally be determined employing simple devices. G et al. [82] measured the thermal potential of thermogalvanic cells using a homemade temperature gradient platform, as shown in Figure 3. Two commercial Peltier chips were used as a thermal and cold stage to generate the temperature difference. A glass substrate was used for electrical insulation. Platinum wires with a diameter of about 0.3 mm were used as electrodes. The two electrodes are connected to the hot and cold ends of the thermogalvanic. To prevent moisture loss during long-term electrochemical measurements, the samples were wrapped with polyacrylate tape. The open-circuit voltage was recorded on an electrochemical station (CHI 660E, Chenhua). Commercial platinum wires with a diameter of about 0.3 mm were used as electrodes. The sample’s location was symmetrical about the center of the temperature field. The temperatures were recorded by thermocouples (Sheffield YET-720L). The thermopower was calculated according to the equation:

(4) $$S_{e}=-\frac{V_{h}-V_{c}}{T_{h}-T_{c}}\frac{d_{2}}{d_{1}}$$

### 2.4. Reaction Kinetics

**Figure 4:**
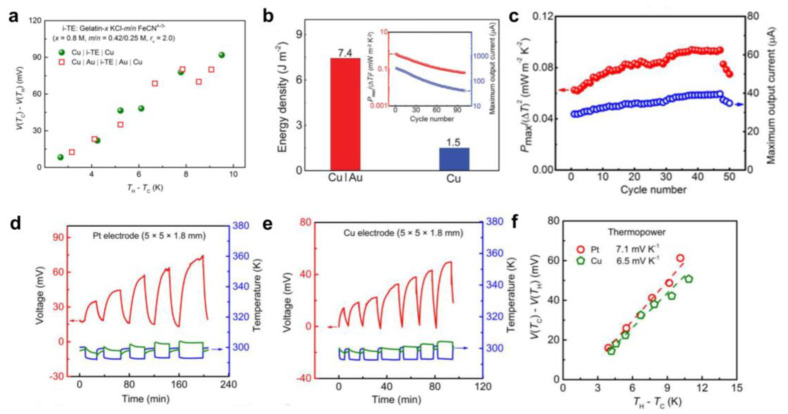
Effect of electrode on the thermopower. (**a**) Thermopower measurement for the as-fabricated i-TE cells Cu|i-TE|Cu and Cu|Au|i-TE|Au|Cu (15 × 15 × 1.8 mm), where i-TE represented Gelatin-0.8 M KCl-0.42/0.25 M FeCN^4−/3−^ (rv = 2.0). Au (40 nm) was coated on the Cu foil with smooth surface by ion sputtering. (**b**) The corresponding total energy density of the initial 50 cycles for i-TE cell with rough Cu|Au (40 nm) and smooth Cu as electrodes. Normalized output power Pmax/(DT)2 and maximum output current of 100 cycles in an i-TE cell (Cu|Au|i-TE|Au|Cu, 15 × 15 × 1.8 mm) are shown in the inset. (**c**) Corresponding Pmax/(∆T)2 and maximum output current varying with the cycle number. Thermopower measurement of i-TE material of Gelatin-0.8 M KCl with (**d**) Pt electrode (Pt|i-TE|Pt, 5 × 5 × 1.8 mm) and (**e**) Cu foil electrode (Cu|i-TE|Cu, 5 × 5 × 1.8 mm). (**f**) Thermopower measurement of the i-TE material (Gelatin-0.8 M KCl) with different electrodes, showing the 7.1 and 6.5 mV K^−1^ for the Pt electrode and Cu electrode, respectively. This suggests that the thermopower is relatively independent of the choice of electrode. The V(T_C_)-V(T_H_) is the voltage difference, while the T_H_-T_C_ is the temperature difference. (Reproduced with permission from [85]. Copyright 2020, AAAS.).

Typically, due to the addition of redox substances, thermogalvanic cells require special electrodes to prevent the oxidation of the electrodes or the interaction of the electrodes with the electrolyte [83,84]. Han et al. found that electrode optimization can increase the output power density of gelatinized thermoelectrochemical cells [85], as shown in Figure 4a,b. Because of the electrode corrosion problem, they coated copper foil (10 mm thick) with gold (40 nm). Since the gold (40 nm) coated copper foil electrode has an expanded surface area, the total energy density for the initial 50 cycles is much higher than that of pure copper foil, as shown in Figure 4c. In most thermogalvanic studies, electrode materials with a high catalytic activity that reduces the charge transfer overpotential are often welcomed, making them ideal tools for checking the performance of a given device. In this work, the researchers found that the thermal power reached 7.1 mV K^−1^ with platinum electrodes compared to copper foil electrodes, which is slightly higher than copper foil electrodes (6.5 mV K^−1^), benefiting from the superior electrical conductivity of platinum electrodes (Figure 4d–f). However, the high cost of platinum makes the exploration of more commercially viable electrode materials an unavoidable option. Almost all recent developments of new thermogalvanic electrode materials are used for aqueous hydrogels based on ferrous cyanide, ferric ions, and iodide ions [86,87]. Thus, using high specific surface area electrodes can significantly increase power.

**Figure 5:**
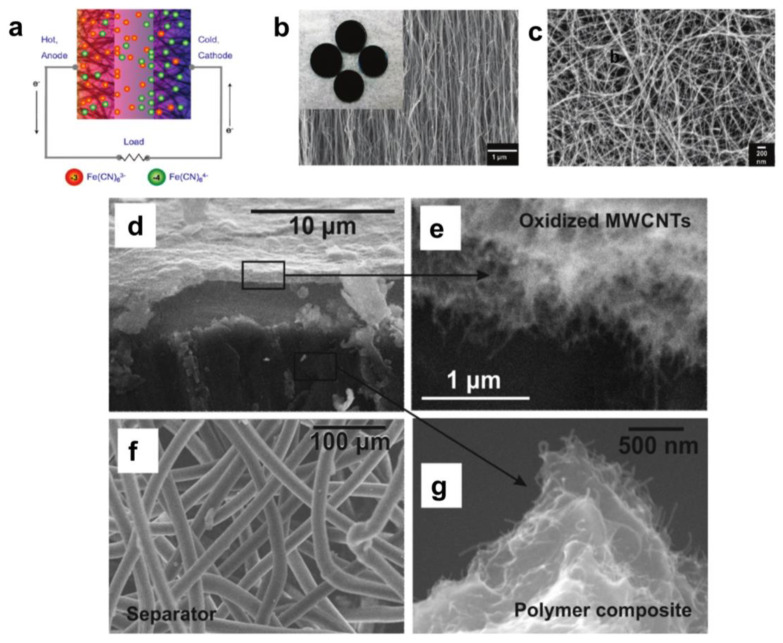
(**a**) Schematic of a thermocell with nanostructured electrodes showing concentration gradients of the ferri-ferrocyanide redox ions during power generation. (**b**) SEM micrograph of vertical MWNT forest. The average MWNT diameter is approximately 20 nm. The inset shows coinlike stainless steel substrates fully coated with vertical MWNTs. Each substrate has a diameter of 2 cm. (**c**) SEM micrograph of MWNT buckypaper. The average MWNT diameter is 10 nm. TEC response in 0.1 M equimolar solution of K_3_Fe(CN)_6_/K_4_Fe(CN)_6_. (Reproduced with permission from [98]. Copyright 2010 American Chemical Society). SEM image of components of the cell: (**d**) electrode structure; (**e**) cover oxidized MWCNTs layer; (**f**) separator; (**g**) fracture of polymer nanocomposite. (Reproduced with permission from [99]. Copyright 2019 IOP Conf. Ser.).

**Figure 6:**
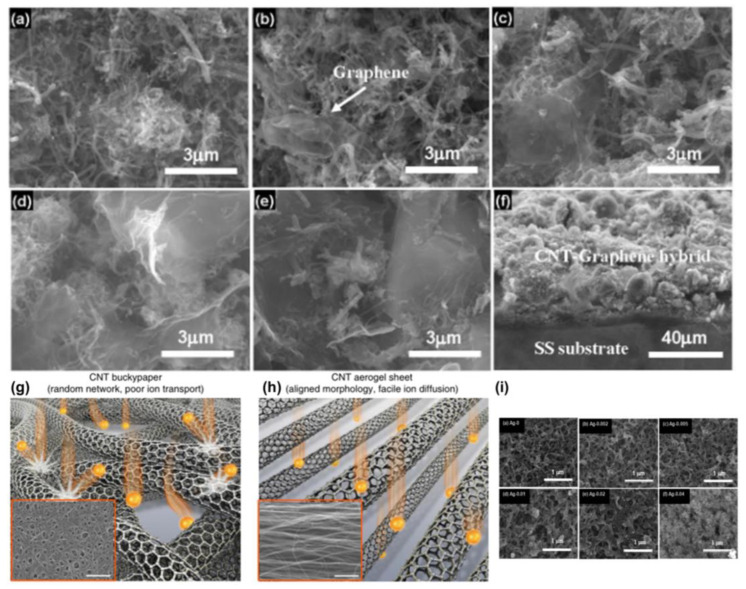
SEM images of the pristine CNTs and CNT–Graphene hybrids. (**a**) The pristine CNTs. (**b**–**e**) The hybrids obtained by adding 0.04, 0.1, 0.2, and 0.4 g·L^−1^ graphenes in the suspension, respectively. (**f**) Cross-section of the CNT–Graphene hybrid. Note: The concentrations of CNTs and Mg^2+^ were kept certain values of 0.1 and 0.03 g·L^−1^, respectively. Carbon nanotube aerogel sheets as high-performance electrodes. (Reproduced with permission from [103]. Copyright 2019 MDPI). (**g**,**h**) Illustrations and SEM micrographs (insets) comparing CNT buckypaper and CNT aerogel electrodes and the relationship of these morphologies to ion transport (scale bars in the insets, 1 mm). MWNT bundling is not shown and only MWNT outer walls are pictured (Reproduced with permission from [104]. Copyright 2016 Springer Nature.). (**i**) SEM images of pristine CNTs and the Ag–MgO–CNTs formed with different concentrations of Ag powder. Pristine CNTs, samples obtained by doping 0.002, 0.005, 0.01, 0.02, and 0.04 g L^−1^ of Ag in the suspension, respectively. Note: the concentration of CNTs and Mg^2+^ were kept constant at 0.1 and 0.03 g L^−1^, respectively (Reproduced with permission from [105]. Copyright 2011 Royal Society of Chemistry).

There are few studies on new thermogalvanics for non-aqueous systems because mass transport is the main limiting factor rather than the charge transfer on the electrode [88,89]. Carbon-based electrodes are gaining increasing attention as a convenient and low-cost alternative to precious metal electrodes [90,91,92,93]. Nanostructured carbon materials, because of their high specific surface area, can increase the number of reaction sites, for example, nanotubes and graphene [94,95,96,97]. In addition, they can have fast electron transfer kinetics for ferricyanide redox pair-based thermogalvanic. Both characteristics can increase the power density of thermogalvanic cells. Hu et al. described a carbon nanotube-based thermogalvanic that utilizes ferricyanide redox coupling and electrodes made of carbon multi-walled nanotube (MWNT) hard paper and vertically aligned MWNT arrays [98], as shown in Figure 5a–c. MWNT was used as the electrode for the thermogalvanic replacing conventional electrode materials, including platinum foil and graphite sheets. The maximum output power of the stagnant cell was 1.8 W m^−2^ at a hot-side temperature of 65 °C and a temperature difference of 60 °C, with an efficiency of 1.4% relative to the Carnot cycle efficiency. Experiments have shown that the performance of MWNT-based thermogalvanics is scalable and that reducing the contact resistance at the MWNT electrode/substrate junction can significantly improve efficiency. This is three times more efficient than conventional thermogalvanic elements using platinum electrodes. As the cost of MWNT decreases, MWNT-based thermogalvanics may become a commercially viable method for obtaining low-level thermal energy. V Shpekina et al. showed that using MWCNT-based polymer composites with improved thermal and electrical conductivity can increase the actual temperature difference between electrodes inside the cell and thus increase the output open-circuit voltage [99], as shown in Figure 5f–h. Thermoelectrochemical cells using polymer electrodes coated with oxidized multi-walled carbon nanotubes and [Fe(CN)_6_]^4−^/[Fe(CN)_6_]^3−^ based electrolytes were developed with current density values exceeding 13 A m^−2^ and with a specific power of 140 mW m^−2^. Calculations based on the temperature dependence of the open-circuit voltage revealed a Seebeck coefficient equal to 0.7 mV K^−1^. This study aimed to measure the thermal conductivity of a flexible, thermally conductive polymer electrode based on an oxidized multi-walled carbon nanotube coating. This study aimed to measure the performance indicators (open circuit voltage, short circuit current, and power output) of a thermoelectric chemical cell based on a flexible thermally conductive polymer electrode coated with oxidized multi-walled carbon nanotubes. Flexible cell bodies with good performance metrics have potential applications as commercial batteries based on thermoelectrochemical cells for harvesting waste heat from various sources such as industrial or geothermal heat, solar heaters or collectors, biomass fermentation, human heat, heating reactors, pipes, or vessels [100,101,102]. In addition, Zhou et al. prepared carbon nanotube graphene (CNT-Gr) hybrids on a stainless steel substrate using the electrophoretic deposition (EPD) technique to prepare thermoelectric chemical cell electrodes, as shown in Figure 6a–f [103]. The TEC performance of these hybrid electrodes was significantly improved compared to the pristine CNT electrodes. These hybrid electrodes were optimized by adjusting the graphene content in the hybrid compounds. The CNT-Gr-0.1 hybrid electrode exhibited the best TEC performance at a current density of 62.8 A m^−2^ and a power density of 1.15 W m^−2^, 30.4% higher than the CNT electrode. The improved TEC performance was attributed to improved electrical and thermal conductivity and the adhesion between the CNT-Gr hybrid material and the substrate. Meanwhile, the relative conversion efficiency of TEC can reach 1.35%. It is shown that the growth of CNT-Gr composite electrodes by the EPD technique may provide a promising method for the practical application of TEC electrodes based on carbon nanomaterials. Hyeongwook et al. devised a method using CNT aerogel sheets as electrodes, removing low-activity carbon impurities that limit electron transfer kinetics, decorating the CNT sheets with catalytic Pt nanoparticles and mechanically compressing the nanotube sheets to adjust conductivity and porosity. The thermogalvanics produced an output power density of 6.6 W m^−2^ at a temperature difference of 51 K, corresponding to a Carnot relative efficiency of 3.95% [104], as shown in Figure 6g,h. The importance of electrode purity, engineered porosity, and catalytic surface in improving thermogalvanic performance is illustrated.

In the electrophoretic deposition (EPD) method, the contact of carbon nanotubes (CNTs) with the substrate is weak, so commercialization as electrodes for thermoelectric chemical cells (TECs) remains challenging. Qian et al. successfully prepared Ag-MgO-CNT nanocomposites on stainless steel (SS) substrates by doping Mg^2+^ and Ag powders in carbon nanotube suspensions using the electrophoretic deposition (EPD) method. Ag-MgO-CNT nanocomposite electrodes showed significantly improved TEC performance due to their higher electrical conductivity, thermal conductivity, and improved adhesion between the composite film and the SS substrate [105], as shown in Figure 6i. The results indicate that the construction of Ag-MgO-CNTs nanocomposite electrodes can effectively enhance the performance of thermochemical cells based on carbon nanotubes (CNTs), which may be a promising approach for energy harvesting using CNT-based thermocells prepared by EPD technology.

**Figure 7:**
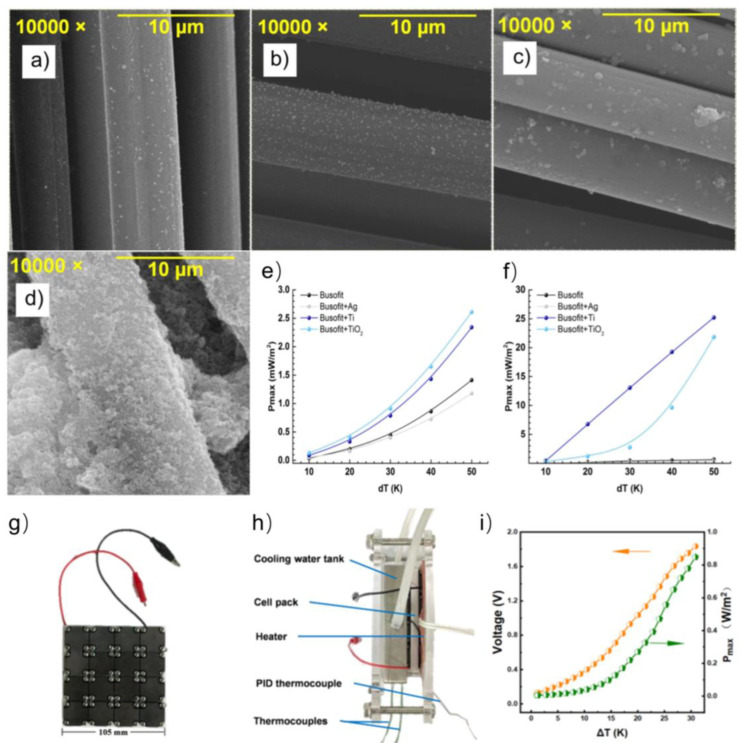
SEM images of the Busofit materials: (**a**) basic; (**b**) with Ag spraying; (**c**) with Ti spraying; (**d**) with infiltrated dispersion of TiO_2_ nanoparticles. Plots of maximum power density versus temperature difference between the hot and cold sides of the cell (**e**) for cells with a salt bridge and (**f**) for cells in the coin cell CR2025. (Reproduced with permission from [113]. Copyright 2021 MDPI). Prototype for BST/FeCN^3-/4-^ thermo–electrochemical cells; (**g**) The prototype consists of 16 cells in series, (**h**) the test system for prototype, and (**i**) thermo–electrochemical performance of the prototype. (Reproduced with permission from [114]. Copyright 2022 Elsevier Ltd.).

However, carbon nanotubes are still limited in industrial production, are extremely expensive, and require the use of polymer binders to fabricate electrode materials [106,107,108]. In contrast, carbon fiber (CF) materials offer significant advantages over polymer additives for carbon nanotubes and carbon black-based electrodes, as they do not require the addition of binding components during electrode fabrication. CF materials are mainly prepared from polyacrylonitrile (PAN) asphalt and rayon [109,110,111,112]. Isotropic bitumen and viscose-based carbon fibers are excellent materials for producing activated carbon fibers with a high specific surface area (>1500 m^2^ g^−1^). They may become the primary materials for liquid and gas adsorption and environmental protection. Therefore, Denis et al. investigated the modification of carbon fiber electrode surfaces using magnetron sputtering by depositing silver and titanium or infiltration injection of titanium oxide nanoparticles to improve the electrodes and its effect on the output power and impedance equivalent scheme parameters of thermoelectric cells [113], as shown in Figure 7a–f. It was found that the nature of the electrode surface modification can increase the internal resistance of the cell by three orders of magnitude. Equivalent scheme parameters and output power density of thermoelectric cells are given as a function of electrode material type. A maximum power of 25.2 mW m^−2^ and an efficiency of 1.37% were observed for titanium and titanium oxide-modified carbon fibers.

Due to the restricted ion migration, gel cells have high intrinsic impedance and low short-circuit current density, which leads to low output power density. Therefore, the practical application of thermoelectric chemical cells is greatly limited. H et al. investigated a new method to increase the selenium content of ferrous cyanide electrolytes by shortening the diffusion length and accelerating the electron transfer, which was achieved by adding micro Bi_0.4_Sb_1.6_Te_3_ powder to [Fe(CN)_6_^3−^\Fe(CN)_6_^4−^] redox couple solution to form a semi-solid gel electrolyte [114], as shown in Figure 7g–i. The BST in the electrolyte acts as a microelectrode and accelerates the electrolyte circulation between the electrodes, leading to a Seebeck coefficient of −4.11 mV K^−1^ and a maximum output power density of 0.99 W m^−2^ when the temperature difference is 30 K. This work provides a novel approach to increase the thermoelectric power and output power density of thermoelectric chemical cells, allowing them to be better used for low-grade thermal energy recovery.

### 2.5. Thermal Transport in Hydrogels

**Figure 8:**
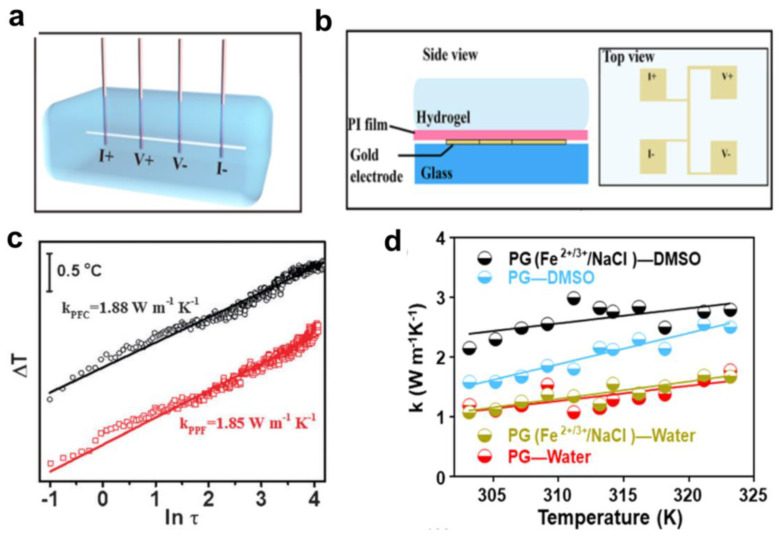
PAAm hydrogel and its experimental setup for thermal conductivity measurement. (**a**) Schematic of 3*ω* method setup for thermal conductivity measurement of hydrogels. A platinum wire is deeply immersed in hydrogels and wired out with four copper probes for applied current and voltage measurements. The four brown rods are copper probes and the white line in hydrogel is the Pt wire. (**b**) Schematic of 3*ω* method for thermal conductivity measurement of hydrogels at different water contents and temperatures. The four Au squares (2 mm in length) on a 1 mm thick glass substrate serve as both the heater and thermometer. (Reproduced with permission from [116]. Copyright 2017 MDPI). (**c**) Thermal conductivities of the thermogalvanic gels at room temperature. (Reproduced with permission from [56]. Copyright 2016 WILEY-VCH Verlag GmbH & Co., KGaA, Weinheim). (**d**) Variation of the thermal conductivity of the gel with temperature by changing different media. (Reproduced with permission from [117]. Copyright 2022 Elsevier Ltd.).

Besides electrical properties, heat transfer in hydrogel thermoelectric devices largely remains to be explored because the hydrogels used in the above fields usually achieve simple action based on ambient temperature sensitivity without the significant involvement of electronic components. Tang et al. reported an experimental study on the thermal conductivity of hydrogels. Polyacrylamide (PAAm) hydrogels were selected as model hydrogels because PAAm is widely used in wearable electronic and thermogalvanic cells. Both experimental and simulation results showed that the cross-linker concentration was a key factor affecting the mechanical properties of the hydrogels, and their thermal conductivity was significantly related to the cross-linker concentration [115]. The 3*ω* method was used in the study to measure the thermal conductivity of the gels [116], as shown in Figure 8a,b. When an AC with an angular frequency of 1*ω* is applied to the two input electrodes, a small voltage signal can be detected across the heater to the other two output electrodes. The voltage with a frequency of 3*ω* is selected and extracted as the signal carrying the thermal effect. Combining the relationship between frequency, voltage, and temperature enhancement, the thermal properties of the hydrogel can be extracted. The thermal conductivity calculated from the 3Ω voltage and frequency is as follows:

(5) $$k=\frac{\alpha V_{1\omega }^{3}}{8\pi lR}\frac{1}{dV_{3\omega }/d\ln\omega }$$

where k is the thermal conductivity of the sample and α is the temperature coefficient of the heater. *V*_3*ω*_ is the 3*ω* voltage of the heater, l is the length of the heater, and *R* is the resistance of the gel before heating by the heater. The feasibility of the measurement device was also verified by testing deionized water at room temperature. The measured thermal conductivity of deionized water was 0.60 Wm^−1^K^−1^, which was in good agreement with the data in the NIST database REFPROP.

There is more than one way to measure thermal conductivity. Yang et al. measured the thermal conductivity of PVA-based gels using the transient hot-wire method [56], as shown in Figure 8c. In our previous study, the thermal conductivity of the gel was determined by the steady-state method [117], as shown in Figure 8d. The equation for calculating the thermal conductivity used in the experiment is as follows:

(6) $$k=\frac{c\times m\times T_{top}}{\Delta t}\times \frac{l}{A\times \Delta T}$$

where *c* is the specific heat (J g^−1^ K^−1^), *m* is the mass of the gel (g), *T_top_* is the initial temperature of the upper surface (*K*), Δ*t* is the heat transfer time to reach the steady state (s), *A* is the surface area of the heat sink end of the gel, and Δ*T* is the temperature change of the upper surface area (*T_end_*-*T_top_*, *K*).

## 3. Selection of Polymer Networks and Encapsulation Methods

**Figure 9:**
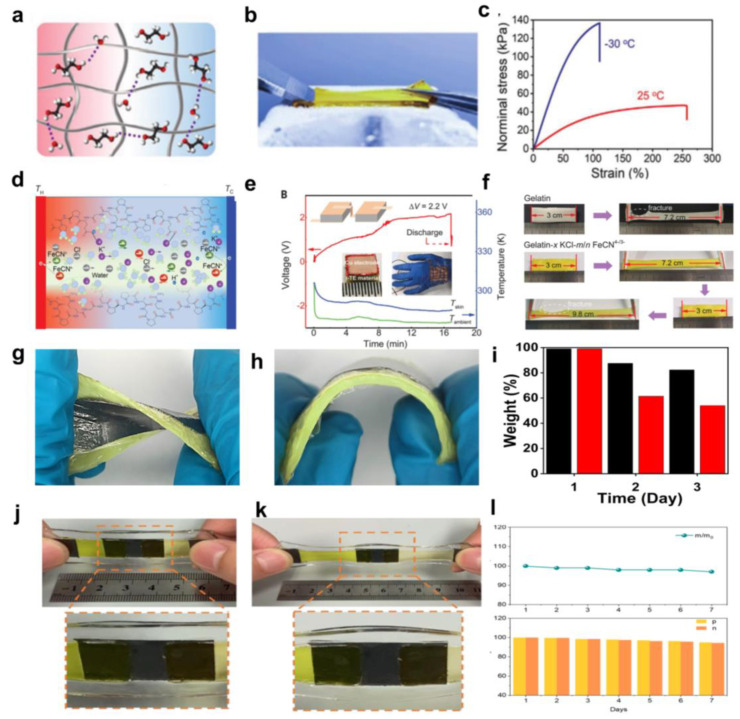
(**a**) Schematic illustration of the organohydrogel thermocell containing crosslinked networks, EG, and water upon a temperature gradient. The additional EG can disrupt the ice crystal lattice to expand the working temperature at subzero temperatures. (**b**) A photograph of the organohydrogel thermocell being stretched on an ice surface of 20 °C). (**c**) The tensile curves of the organohydrogel thermocell in ambient (25 °C) and cold (−30 °C) environments. Giant thermopower of i-TE materials (Reproduced with permission from [82]. Copyright 2021 Wiley-VCH GmbH). (**d**) Schematic figure of the diffusion, redox reaction, and interaction of the ions in the as-fabricated i-TE materials of Gelatin-x KCl^−m/n^ FeCN^4–/3–^ under the temperature gradient. (**e**) The voltage generated from a proof-of-concept flexible i-TE wearable device with 25 unipolar elements (Cu | i-TE | Cu, 5 × 5 × 1.8 mm, smooth Cu foil) in series worn on the back of the human hand. (**f**) Tensile test of the i-TE material of Gelatin-0.8 M KCl-0.42/0.25 M FeCN^4–/3–^ (rv = 2.0) compared with pure gelatin. (Reproduced with permission from [85]. Copyright 2020, AAAS.). (**g**,**h**) Photographs of the gel patch that was twisted and bent. (**i**) Dehydration of the gel thermoelectric patch (Black)and commercial fever-reducing patch (Red). (Reproduced with permission from [118]. Copyright 2021 American Chemical Society). (**j**,**k**) Photos of graphite electrode connected p-n cell in original (**left**) and stretchable (**right**) state. (**l**) The water retention rate and Seebeck coefficient retention rate of p- and n-type hydrogel encapsulated by VHB tape. (Reproduced with permission from [55]. Copyright 2022 Wiley-VCH GmbH).

From the previous research, existing thermoelectric materials encounter limited mechanical adaptability, unsustainable operation, and concerns about environmental safety. To this end, the researchers propose a gel device for low-grade heat harvesting that achieves excellent quasi-solid-state properties through the cooperation of polymer networks. With the research on gels, the attention on hydrogels has gradually shifted from basic research to rich functionalization. The current hydrogels still have relatively simple structures and poor environmental adaptability, which greatly limit their practical applications in complex environments. Using the complementary organic solvents, thermoelectric gels have gained more excellent environmental adaptability. Furthermore, the heat resistance, switchable mechanics, adaptive wettability, and compatibility of opposite components of the organohydrogel clearly emphasize its promising applications. For example, Gao et al. chose the commonly used acrylamide (AM) as the primary polymer monomer by introducing an azeotropic effect in aqueous thermogalvanics to interfere with the strong hydrogen bonding that the polymer chains remain entropically elastic below zero degrees [82], as shown in Figure 9a–c. It remains more than 110% when the temperature decreases to −30 ℃. This is the first generation of soft-stretch thermogalvanics that can operate in extremely cold environments. The operating mechanisms and chaotic effects studied in this research will also inspire solving the challenges faced by stretchable, wearable, and portable devices that require continuous power at high altitudes, at the North and South Poles, and even in outer space. In addition, Han et al. demonstrated a huge thermoelectric effect in gelatin-based thermogalvanic materials, thoroughly combining the thermogalvanic effect of redox ion pairs (FeCN^4–/3–^) and the thermodiffusion effect of ion providers (KCl) to obtain thermal powers up to 12.7~17.0 mV K^−1^ [85], as shown in Figure 9d–f. Due to the excellent quasi-solid state nature of gelatin, the concept of scaled integration of 25 P-type components was quickly implemented, validating a flexible thermoelectric wearable, integratable device, resulting in an output of high voltage up to 2.2 V. This thermoelectric cell can work for long periods of time in a quasi-continuous thermal charge/discharge mode and also in a continuous mode, providing a maximum energy density of 12.8 J m^−2^. This work provides an extremely promising approach to achieve a wireless and passive supply of flexible sensors for the Internet of Things, demonstrating the promise of using ions with synergistic effects as energy carriers in gelatinized thermoelectric energy conversion.

Unfortunately, while the polymer network fit effectively binds the electrolyte, water dissipation from the aqueous hydrogel is still an unavoidable problem. Therefore, further encapsulation of the thermogalvanic cell plays a key role. Our previous work described a wear-resistant thermogalvanic hydrogel with a polyvinyl alcohol hydrogel electrolyte encapsulated in a Polyurethane (PU) film [118], as shown in Figure 9g–i. PU is a non-toxic and harmless environmental protection material because it is harmless to human skin and is widely used in clothing fabrics, medical and health practices, leather, etc. In addition, while effectively transferring heat from human skin, it has the advantages of good elasticity, high lightness, and free penetration of human sweat through the film. Although the thickness of the film is extremely thin (0.012–0.035 mm), it has physical properties unmatched by other materials. The use of PU film effectively prevents water loss from PVA hydrogel, which is conducive to the long-term work of thermogalvanic devices. Xu et al. used commercial VHB tape from the 3M Company for encapsulating Π-type thermogalvanic machines to prevent performance degradation due to dehydration [55], as shown in Figure 9j–l. VHB tape is composed entirely of polyacrylate, which is highly resistant to heat, UV light, and chemical reagent damage due to the C-C bonding of the polyacrylate backbone, a carbon–carbon single-bonded backbone. The researchers found that potassium hexacyanoferrate in P-type hydrogels will generate Prussian blue precipitation after it encounters ferrous ions in N-type hydrogels. The VHB tape also acts as a spacer to prevent mixing the two electrolytes. 

Furthermore, the matched stretchability of the VHB tape and hydrogel ensures deformability and prevents the possibility of delamination during deformation. The combination of the hydrogel, electrode, and spacer bar did not slide relative to each other in the horizontal stretching test, thus proving the deformation resistance of the device. This work demonstrates the potential of hydrogel-based thermogenic cells in everyday wearable power systems.

## 4. Integration of Thermoelectric Hydrogels

As aforementioned, theoretically, quasi-solid thermogalvanic hydrogels have infinite possibilities by combining different polymer networks and redox species. Polymer networks assist in the formation of hydrogels in several ways and modulate their structure and properties, such as hydrogen bonding interactions, micro crystallization, and the cross-linking method. In this section, we provide a comprehensive introduction to the integration of thermogalvanic hydrogels and application scenarios.

### Cell Connection Methods

**Figure 10:**
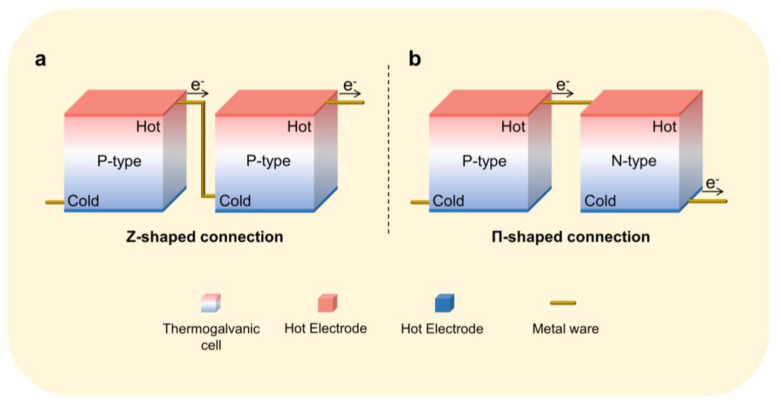
(**a**) Z-shaped connection for a cell. (**b**) Π-shaped connection for a cell.

In practical applications, the voltage output of a single cell is limited by the thermoelectric power and temperature difference; therefore, integrating multiple thermogalvanics in a single device by connecting them in series to produce a real voltage (>1 v) is the most common method. There are two kinds of integration methods: one is the Z-shaped connection for a cell containing only one type of thermogalvanic (n-type or p-type) (Figure 10a), and the other is the Π-shaped connection for a cell containing two types of thermogalvanic (n-type and p-type) (Figure 10b). For the thermogalvanic, one which loses electrons at the hot electrode is usually defined as p-type. Otherwise, a thermogalvanic that gains electrons at the hot electrode is defined as n-type. In simple terms, the hot electrode is higher than the cold electrode for a p-type thermogalvanic and vice versa for an n-type thermogalvanic. 

**Figure 11:**
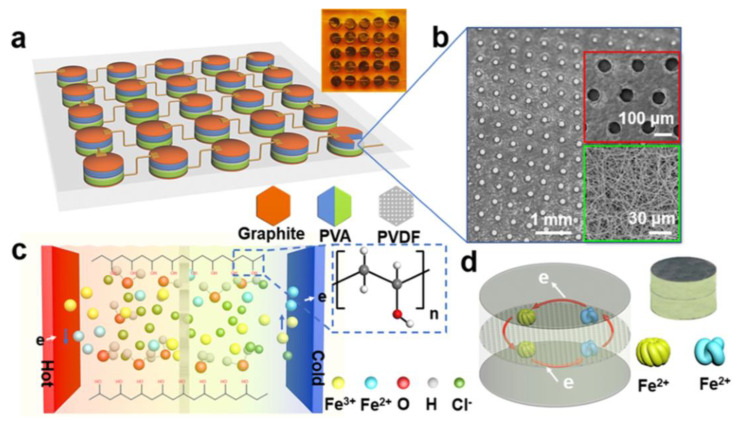
Thermocell working principle. (**a**) Integrated gel-based thermocell. The PVA gel was sandwiched between two flexible substrates (polyimide, PI), the porous PVDF diaphragm is inserted into the two gels, and the device is bridged by a tandem structure. The top right inset is a photograph of the integrated device (scale bar: 10 mm). (**b**) SEM photograph of the porous PVDF diaphragm. (**c**) Schematic representation of the partial contribution of the thermal potential in a complex system containing the structures of iron ions, ferrous ions, chloride ions, hydrogen ions, water molecules, and polyvinyl alcohol molecules. (**d**) Schematic diagram of the operating mechanism of a single thermoelectric gel and a physical photograph of the individual gel (scale bar: 10 mm). (Reproduced with permission from [118]. Copyright 2021 American Chemical Society).

One of the benefits of the Z-shaped connection is that by using the highest conversion efficiency thermoelectric cells, the integrated device can achieve maximum power output. Our current research demonstrated a module based on a Z-shaped connection of 25 units (PVDF thermal barrier enhanced thermogalvanic, boosted Se from 0.58 to 0.79 mV K^−1^) [118], as shown in Figure 11. This PVA-based hydrogel thermoelectric part has excellent flexibility, which can be readily pasted on the body to obtain low-grade body heat. Due to the good biocompatibility and mechanical strength of the PVA gel, the gel thermoelectric cell is non-toxic and has excellent flexibility; due to this excellent performance, the device can be easily attached to the human body for obtaining low-grade body heat. However, Z-shaped connections complicate the device integration process, leading to unstable contacts that can introduce considerable contact resistance between the electrodes and the Z-conductor. 

**Figure 12:**
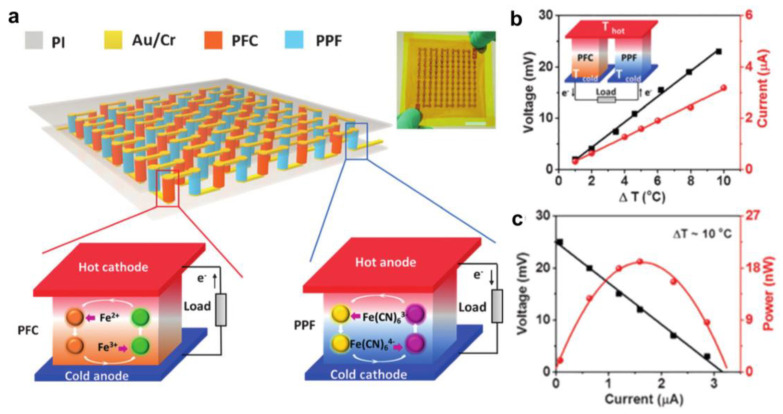
The integrated gel-based thermocell. Both the PFC and PPF gels were sandwiched between two flexible substrates (polyimide, PI). With alternating top and bottom interconnections, the PFC and PPF gels are connected sequentially in series. The magnified insets illustrate the operation mechanism of the gel-based thermocell. At a certain temperature difference, the thermo–voltage polarity of PFC and PPF is exactly reversed. The top right inset is a photograph of the integrated device (scale bar: 2 cm). (Reproduced with permission from [56]. Copyright 2016 WILEY-VCH Verlag GmbH & Co., KGaA, Weinheim).

**Figure 13:**
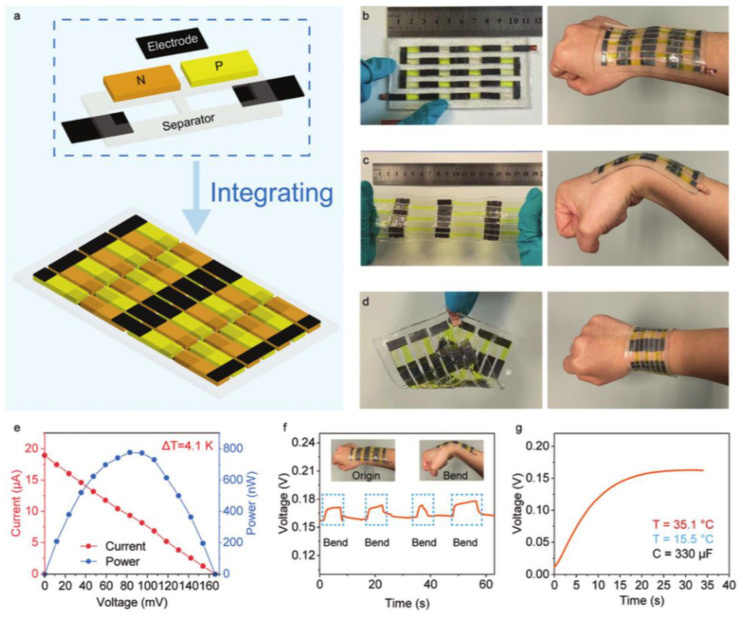
Wearable hydrogel thermocell for energy harvest. (**a**) Illustration of the integration of stretchable thermocell device. (**b**–**d**) Photos of the thermocell device attached to the human body undergoing different deformation states: (**b**) original state, (**c**) stretch-and-bend deformation, (**d**) fit to the curved surface. (**e**) The thermoelectric performances of a device integrating 14 pairs of p-n cells. (**f**) Voltage-time curve of thermocell attached to the wrist during deformation. (**g**) Voltage-time curve of a capacitor of 330 µF charged by the thermocell utilizing human body heat while the ambient temperature is 15.5 °C and the hot end temperature is 35.1 °C. (Reproduced with permission from [55]. Copyright 2022 Wiley-VCH GmbH).

In contrast, Π-shaped connections simplify manufacturing and make wire connections between multiple cells simpler and more reliable in favor of large-scale integration. Zhou et al. demonstrated a PVA-based thermoelectric module integrating 59 p-type cells and 59 n-type cells connected by Π-shaped [56], as shown in Figure 12. The integrated wearable device produces an open-circuit voltage of about 0.7 V and a short-circuit current of about 2 μA using body heat, reaching a maximum output power of about 0.3 μW. Recently, Xu et al. have established a flexible, stretchable wear-resistant thermogalvanic of AAM hydrogel. It can be used for heat harvesting at joints during human movement and is capable of charging a 330 μF commercial capacitor, as shown in Figure 13 [55]. In this work, PAAm hydrogels were selected as the intrinsic hydrogel matrix for thermogalvanics because of their excellent mechanical properties. In contrast, classical redox pairs K_4_[Fe(CN)_6_]/K_3_[Fe(CN)_6_] and Fe(ClO_4_)_3_/Fe(ClO_4_)_2_) with similar thermoelectric properties were selected as n/p-type ion pairs, respectively. The authors captured a temperature difference of 4.1 K by attaching a device with 14 pairs of p-n thermogalvanics to a human joint; this minor temperature difference allows the device to have a voltage output of 0.16 V. This work demonstrates the potential of hydrogel-based thermogenic cells in everyday wearable power systems, in which low cost, good output performance, structural simplicity, stretchability, and skin-like appearance are advantages. Note that due to the lack of high thermal efficiency n-type cells, devices integrated by Π-shaped connection remain low performance.

**Figure 14:**
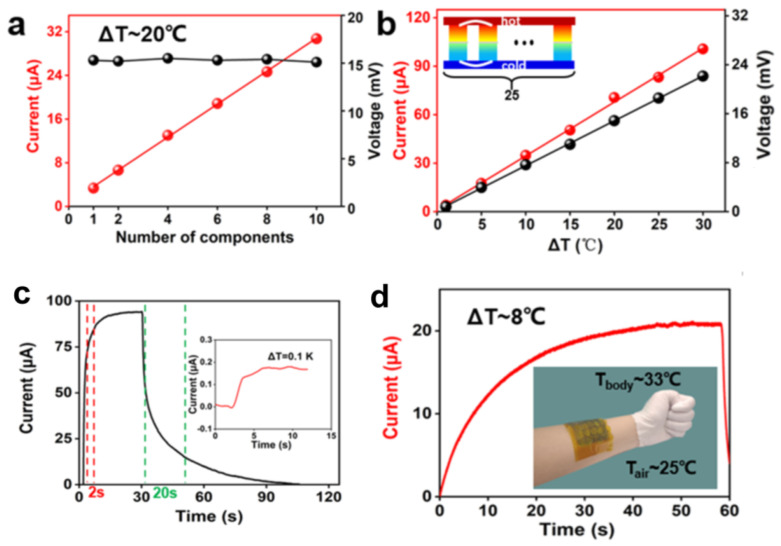
Thermoelectric performance of the integrated device under certain temperature difference conditions. (**a**) Output current and voltage of an integrated device consisting of different numbers of thermocells connected in parallel. (**b**) Current and voltage versus temperature difference for 25 parallel units. The illustration is a schematic diagram of 25 batteries in parallel. (**c**) Magnified current response curve vs. time. Inset: current−time curve under a temperature difference of 0.1 K. (**d**) Current output when the integrated patch device is pasted on the arm. Inset: photograph of the wearable thermocell on the arm. (Reproduced with permission from [118]. Copyright 2021 American Chemical Society).

In addition, the current can be effectively enhanced by connecting the devices in parallel. In our current study, we have displayed the parallel and series integration of 1, 2, 4, 6, 8, and 10 gel components, which provides a feasible pathway for the large-scale integration of thermal cells. The output currents and voltages of the integrated devices composed of different numbers of cells in series are shown in Figure 14a. The voltage remains relatively stable, while the current shows a linear increase with the increasing cells. The device can deliver an output current of about 31 μA in parallel when the integration number of units is 10. To further improve the output performance, 25 gel cells (2 mm high and 6 mm diameter) were integrated into the parallel, as shown in Figure 14b. At a temperature difference of 30°C, the parallel output current is up to 100 μA. As a sensor, when the gel patch is close to the heat source, the current output reaches 80% of the maximum value within 2 s and drops to 20% of the maximum value within 20 s after leaving the heat source. The inset of Figure 14c indicates a temperature detection limit of 0.1 K. As shown in Figure 14d, when the integrated device is affixed to the arm, a short-circuit current of about 20 μA is generated at an ambient temperature of 25 °C. This work provides a new avenue for body heat harvesting and smart medicine based on wearable or implantable electronic devices.

## 5. Potential Applications for Thermogalvanic Hydrogel Devices

Hydrogels are known to be a newly emerging soft material, and researchers have been working to expand the potential applications of polymer network hydrogels in flexible electronic devices. Hydrogels with excellent mechanical toughness and elasticity have been developed as a versatile platform for integrating various micro-devices, such as body temperature sensors, skin pressure sensors, and other biologically relevant aspects, without additional support substrates [119]. Sensors, in general, are devices that are used to simulate the detection and response to external stimulus signals, an inherent function contained in human sensory organs. The critical role of a sensor is to convert a physical or chemical stimulus into another form of energy. The hydrogels used in traditional sensors are mainly used to detect signals from the human body. The extremely high water content makes the physical properties of the thermogalvanic hydrogels similar to those of skin tissue, offering good biocompatibility and excellent prospects for thermal sensor applications [120]. Wearable electronic devices based on thermogalvanic hydrogels are considered to play an important role in medical diagnostics, biosignal detection, and other related aspects. 

### 5.1. Body Temperature Sensors

**Figure 15:**
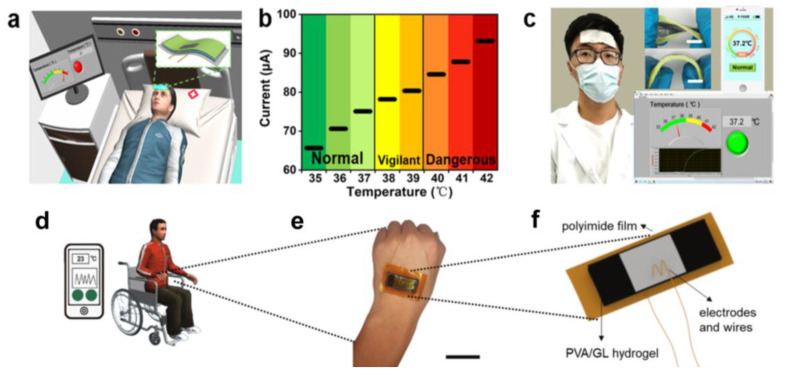
Application demonstration of wearable medical electronics. (**a**) Panoramic view of the gel-based thermoelectric patch for body temperature monitoring. (**b**) Relationship between current and body temperature in the three typical regions. (**c**) Body temperature monitoring and the corresponding temperature signal displayed on terminals. Inset: photographs of the gel patch that was twisted and bent (scale bar: 1 cm). (Reproduced with permission from [118]. Copyright 2021 American Chemical Society). Application demonstration of self-powered temperature monitoring using the PVA/GL thermogalvanic hydrogel. Application demonstration of self-powered temperature monitoring using the PVA/GL thermogalvanic hydrogel. (**d**) Panoramic view of the gel-based patch for ambient temperature monitoring. (**e**) Photograph of a gel patch worn on the back of the hand. Scale bar (5 cm). (**f**) Schematic diagram of PVA/GL gel patch. (Reproduced with permission from [121]. Copyright 2022 Royal Society of Chemistry).

Regarding applications, in order for a thermogalvanic hydrogel to function as a self-driven sensor, the hydrogel must respond to an external stimulus and generate a voltage or current signal. Recently, we have developed a gel electrolyte-based thermo-current generator prepared using Fe^3+^/Fe^2+^ as a redox pair, which has not only moderate thermoelectric properties but also excellent flexibility. Considering that the gel has a good temperature response, a self-powered body temperature monitoring system was established by conformally attaching it to the forehead [118], as shown in Figure 15a–c. Due to its excellent flexibility, our gel patch can withstand bending and twisting. When the patch is applied to the forehead, the receiving terminal, including the computer and cell phone, can display the temperature in real-time. We also monitored the current profile and temperature displayed on the terminal in three body temperature states. In addition, we made a self-driven body temperature monitoring device with a fast response rate; when the gel patch is close to the heat source, the current output reaches 80% of the maximum value within 2 s, and the output drops to 20% of the maximum value within 20 s after leaving the heat source. Meanwhile, gel patches with high specific heat capacity can effectively cool fever patients. This work provides a new avenue for body heat harvesting and smart medicine based on wearable or implantable electronic devices.

Aqueous dispersion media inevitably freeze at sub-zero temperatures due to strong hydrogen bonding, and conventional gel electrolyte thermal cells are limited in their applications. The polymer network is limited by the reduced entropic elasticity, which severely affects the mechanical and thermoelectric properties of the thermal cell. As a result, there are only very few cases where stretchable power sources can operate in extreme environments, such as outdoors in extreme weather. Li et al. weakened the hydrogen bonds in the hydrogels by introducing a chaotropic agent, which not only made the polymer chains entropically elastic at temperatures below zero degrees but also increased the solubility of the electrolyte by using H_2_O/GL (glycerol) as a binary solvent, thereby increasing the thermal potential [121], as shown in Figure 15d–f. As a result, the prepared thermogalvanic hydrogel can exhibit a wide operating temperature range (−20 to 80 °C) as well as excellent resistance to desiccation, which can potentially adapt to harsh temperature environments. Based on its sensitive response to temperature changes, this self-powered temperature monitoring system has been shown to be able to actively monitor abnormal temperatures in the human body.

### 5.2. Environmental Temperature Monitoring

**Figure 16:**
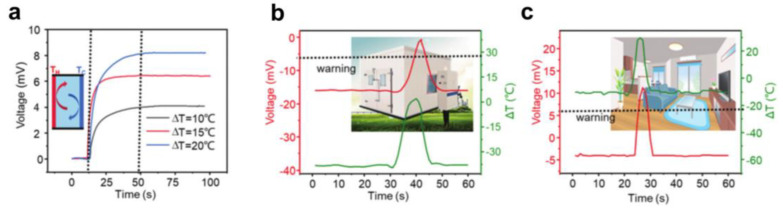
Application demonstration of self-powered temperature monitoring using the PVA/GL thermogalvanic hydrogel. (**a**) The response time of the H-window. Voltage response and DT (Tin–Tout) of a refrigeration storage unit (**b**) and house (**c**). (Reproduced with permission from [121]. Copyright 2022 Royal Society of Chemistry).

In addition, ambient temperature monitoring is also a suitable application [121]. LI et al. also constructed a smart window by embedding a micro PVA/GL hydrogel and placing it at −20 °C, as shown in Figure 16a. Since the size of the implanted hydrogel is negligible, the window still works properly. Assuming a relatively constant room temperature, the hydrogel can generate a voltage signal corresponding to the outdoor temperature. In this practical application, the output voltage signal of the window is plotted over 24 h. The voltage profile shows an almost identical waveform to the temperature difference, indicating that the output voltage can accurately track the outdoor temperature.

Based on this, the authors also demonstrate two additional simulation scenarios. The temperature inside a conventional refrigerated warehouse is typically maintained in the −18 °C range. Using this smart window to track the temperature difference between the internal and external temperatures, it is determined whether the freezer is operating properly based on the voltage signal generated. If the voltage exceeds 8 mV, it indicates that the internal temperature of the reefer has risen above 0 °C (assuming the outdoor temperature remains around 20 °C), resulting in a warning, as shown in Figure 16b. In addition, the smart window can track the room temperature in daily life. If the sensed voltage exceeds 8 mV, it indicates an abnormally high indoor temperature and can activate a fire alarm, as shown in Figure 16c. Overall, the smart window can be widely used for self-powered temperature monitoring in various scenarios. With a wide operating temperature range, thermogalvanic hydrogel is a solid step toward practical self-powered temperature monitoring.

### 5.3. Solar Energy Collection

**Figure 17:**
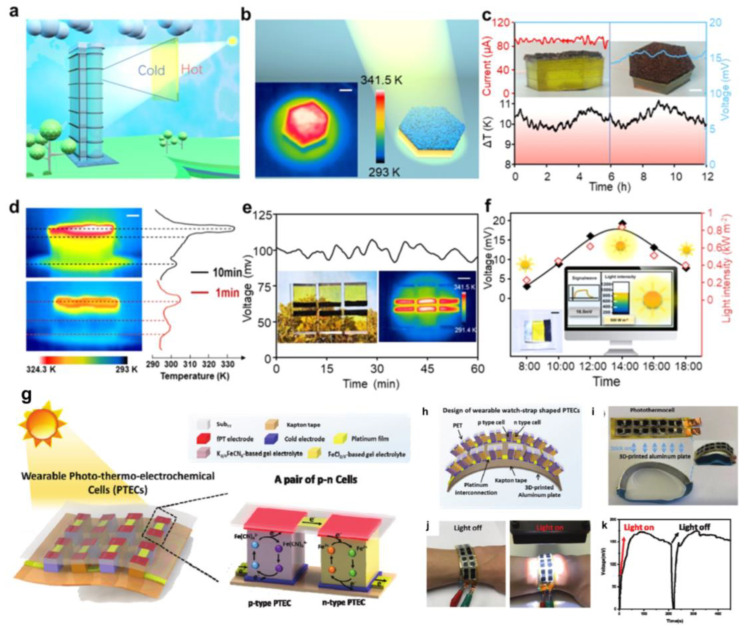
Demonstration of harnessing solar energy. (**a**) Panoramic view of a PG gel-based window for thermal energy harvesting. (**b**) Schematic diagram of light absorption by the porous PDMS sponge. The inset shows the temperature distribution under the simulated sunlight illumination (scale bar: 1 cm). (**c**) Output current (red), output voltage (blue), and temperature difference (black) between the two ends of the gel. Inset: the PG gel with the surface covered by a light-absorbing sponge, front view (**left**), top view (**right**) (scale bar: 10 mm). (**d**) The temperature distribution of a PG gel device with an optical density of 1 kW m-2 under 1 min and 10 min of light. (**e**) The operating open circuit voltage for a real window model. Inset: a photo of a thermoelectric window placed in outdoor sunshine (**left**) and the corresponding infrared image at a light intensity of 600 W m^2^ (**right**) (scale bar: 20 mm). (**f**) Output voltage and light intensity change throughout a day. Inset: light intensity detection system and physical photos of the device (scale bar: 1 cm). (Reproduced with permission from [117]. Copyright 2022 Elsevier Ltd.). (**g**) Structural illustration of the integrated wearable PTEC device, where p-type and n-type devices are connected in series to form a pair of p–n cells and then multiple repeat p–n unit cells were connected in series. Flexible and wearable p–n cells. (**h**) Schematic illustration; (**i**,**j**) photos and (**k**) open circuit voltage of the flexible watch-strap shaped PTEC array harvesting solar energy [122]. (Reproduced with permission from [120]. Copyright 2022 Wiley-VCH Gmbh).

Solar energy is an inexhaustible energy source for developing environmentally friendly energy technologies. Solar thermal technology is a direct solar energy acquisition strategy that allows for the highest conversion efficiency and a wide range of applications. In our previous work, strong hydrogen bonding was inhibited by inducing DMSO solvent, which not only makes the polymer chains tropically elastic at temperatures below zero degrees, effectively improving the conductivity in the low-temperature state, but also forms microcrystalline regions in the cross-linked structure, which facilitates the transparency of the thermal cell. To demonstrate the potential application of low-level harvesting heat induced by solar radiation, we established a PG gel-based thermal energy harvesting window [117], as shown in Figure 17a–f. The temperature difference between the two ends of the gel is generated by an implanted light-absorbing sponge. A PG gel electrolyte with a porous structure and a solar absorber sponge are assembled into a cell called PGP. The prepared PDMS sponge with an interconnected network structure, and a built-in rough surface provides an ideal framework for immobilizing photothermal nanomaterials and light scattering. Thanks to the carbon black, the PDMS sponge (thickness of 3 mm) exhibits very small optical transmittance and reflectance (<0.1%) in the visible and near-infrared spectra, indicating that the PDMS sponge has significant light absorption. The temperature difference at the PG thermogalvanic interface with and without the light-absorbing sponge confirms a significant solar thermal conversion (Figure 17d). The PDMS sponge is heated to 340 K for ≈ 60 s. With exposure to sunlight for more than 12 h, the maximum temperature difference was about 11.2 K, the open-circuit voltage was up to 16.6 mV, and the short-circuit current exceeded 100 μA. With the outstanding features of easy mass preparation, excellent flexibility/modularity, and high transparency, we constructed a true thermoelectric window with outdoor solar radiation of about 600 W m^−2^ (ΔT ≈ 10 k). The solar absorber and heat sink are mounted on both sides of the window frame so that the whole thermoelectric device does not interfere with the light-harvesting. Due to its good photovoltaic conversion capability, the PGP can be used for self-powered solar intensity monitoring. The output voltage of the device is converted to real-time solar intensity and fed back to the simulation platform. Overall, this PGP device offers the possibility to power the sensor with the required voltage in a sunny environment, thus improving the overall combined utilization of solar energy. This integrated recyclable thermogenic cell and a deformable component may open new avenues for the joint production of low-level thermal energy harvesting and power, readily utilizing widely available waste heat and green energy sources. In the work of Liu et al., wearable photothermal electrochemical (PTEC) cells were investigated, in which p- and n-type single PTEC devices were optimized and successively realized the effective series connection of p-n device arrays for solar energy collection applications [122], as shown in Figure 17g–k. First, the flexible PT material PEDOT: PSS was selected as the solar absorber and thermal electrode for PTEC. Second, the PEDOT: PSS/PET fPT electrode/Sub_FT_ combination was assembled into a PTEC device, and the performance was tested in p-type and n-type gel electrolytes, which provided the best performance among devices with other fPT electrode/Sub_FT_ combinations. Meanwhile, a pair of p-type and n-type PTECs with comparable output currents (p-n cells) was coupled to achieve a power density equal to the sum of the p-type and n-type half-cells, indicating the low internal resistance of the electrode materials and the effective matching of the p-n cells. Subsequently, an array of 18 pairs of p-n cells was fabricated that could achieve high voltages under solar simulator illumination and charge the supercapacitor to more than 250 mV. To enable wearable applications, flexible PTEC devices are preferred. Although the electrodes and electrolytes are highly bendable, the large deformation (mainly stretching) of the top electrode compared to the bottom electrode makes it difficult to conform to the bent surface. For this reason, PTECs have a strap shape that conforms to previously developed designs. Meanwhile, the flexible PTEC array device is affixed to a 3D-printed thermally conductive aluminum bracelet to maintain relatively the same temperature of all bottom electrodes. We demonstrate that the flexible strap-shaped PTEC array device can be easily worn on the human arm and that the device can be charged up to approximately 160 mV (eight pairs in series) once exposed to light. The demonstration shows the promise of flexible PTEC arrays for wearable applications. The recorded solar-driven voltage output suggests that the device may be suitable for practical applications. Furthermore, it is noteworthy that the materials used in this work (including fPT electrodes, cold electrodes, and gel electrolytes) are readily available and the fabrication process can be easily implemented, which makes it promising for large-scale commercialization. Thus, the wearable PTECs developed in this work will provide new insights into the future use of solar energy to power next-generation wearable electronics at the commercial level.

### 5.4. Body Heat Harvesting

**Figure 18:**
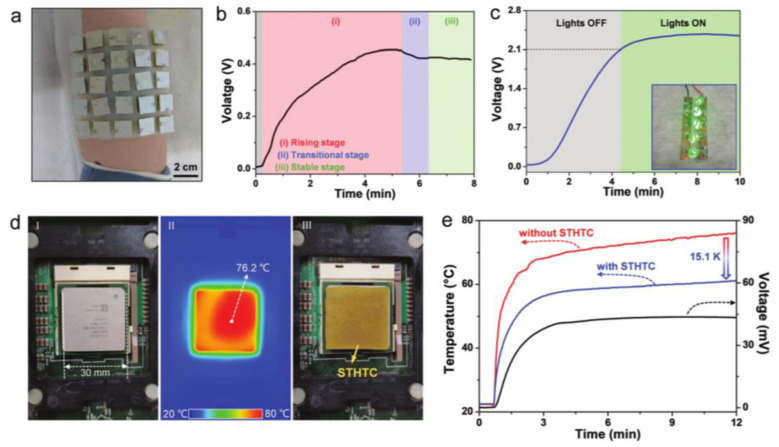
Practical applications of the STHTC. (**a**,**b**) The optical image of the 5 × 5 STHTC array device being fixed on an arm for harvesting human thermal energy (**a**) and corresponding voltage output (**b**). (**c**) The output voltage of the array device with the ice-box surface as the cold source and a square heating plate with a stable temperature of ~36.5 °C as the hot source for simulating human temperature, and the inset indicates that the produced electricity can directly light up five green LEDs. (**d**) The optical image of a normal working CPU (I) and corresponding infrared image with the maximum temperature of 76.2 ℃ (II), a STHTC placed on the surface of the CPU for device cooling and power generation (III). (**e**) The temperature curves of the CPU and corresponding output voltage, indicate the obvious temperature reduction of ~15.1 K when the CPU was covered with the STHTC. (Reproduced with permission from [123]. Copyright 2011 Royal Society of Chemistry).

Zhou et al. described the fabrication process of K^3/4^[Fe(CN)_6_] porous gel electrolytes combined with Pt electrodes as p-type thermogalvanic (PAM-K^3/4^Fe(CN)_6_), as shown in Figure 18a–c [123]. The optimized lyophilized porous gel electrolyte was selected for further study. The researchers also introduced GdmCl into the K^3/4^[Fe(CN)_6_] thermocell and measured the thermoelectrochemical performance at different GdmCl concentrations, temperature differences, and electrode temperatures. Finally, the lyophilized porous thermogalvanic hydrogel was also combined in series with a PAM-Fe^2+^/Fe^3+^ thermogalvanic, which was integrated with a Pt electrode to form an n-type cell (PAM-FeCl_2/3_-HCl). To achieve higher voltage output, paired p-type and n-type TECs (p-n cells) were fabricated as a demonstration device. To collect body heat through wearable thermogalvanic, a flexible band design conforming to the skin surface is ideal and was therefore designed and prepared. The ambient air electrodes were slightly separated from the body heating electrodes to achieve the required curvature for fabricating the device. To demonstrate the fabricated device, one researcher wore a single-band thermogalvanic array (9 pairs of p-n thermogalvanic) on his wrist, which can charge a 100 μF supercapacitor during long-term wear and power a LED when combined with a voltage intensifier. This demonstration illustrates the potential effectiveness of body heat to power certain self-powered epidermal electronics. Zhang et al. designed a polyacrylamide/(sodium alginate) (PAAm SA) dual network stretchable thermoelectric flowing gel thermal cell (STHTC) for a ferrous/ferricyanide [Fe(CN)_6_]^4−^/[Fe(CN)_6_]^3−^ion redox couple based on ferrous/ferricyanide [Fe(CN)_6_]^4−^/[Fe(CN)_6_]^3−^ ions [123], as shown in Figure 18. With guanidinium that was ion-induced [Fe(CN)_6_]^4−^ in order to enhance the thermo-current effect, STHTC exhibited an average thermal power, conductivity, and stretching of about 4.4 mV K^−1^, 10.5 S m^−1^, and 540%, respectively. A record 1780 μW m^−2^ K^−2^ of Pmax ΔT^−2^. This is approximately three times the highest reported value based on individual thermo–current effects. As a stable heat source, the human body can continuously output low-level thermal energy, and it is expected to develop self-powered wearable devices by harvesting human thermal energy. To illustrate the practical value of STHTC, the researchers designed a 5×5 STHTC array device based on 25 STHTC cubic blocks. The STHTC blocks are connected in series and integrated into a silicone mold, which demonstrates the good flexibility and bendability of the STHTC array device. The output voltage of the array device fixed on the arm for collecting human thermal energy can be stabilized at 0.42 V. For practical applications, the STHTC array device is placed on the surface of a refrigerator (~0 °C) for simulating a winter environment, and a square heating plate with a stable temperature of ~36.5 °C is placed on top of the device for simulating human body temperature. At a steady state, the device can provide a high voltage of ~2.3 V. Five green LEDs can be lit when the voltage exceeds 2.1 V, which shows the potential application of STHTC in daily life by harvesting low-level heat energy. In addition, the heat generated by the rapid increase in the total power consumption of electronic devices significantly reduces operational performance and exacerbates electronic device failures.

### 5.5. Heat Generation Device Heat Harvesting

The central processing unit (CPU) generates a lot of heat during normal operation, which severely reduces the stability and operating speed of the computer. When the operating temperature is above 70 degrees Celsius, CPU performance decreases by 10% for every increase of 2 degrees Celsius. The temperature of a normal operating CPU can be as high as 76.2 °C. Achieving effective device cooling to improve device performance is critical. STHTC contains a large amount of water, which can carry away heat through evaporation. Therefore, it is feasible to use STHTC to cool electronic devices and even generate electricity. To demonstrate the practicality of cooling CPUs, researchers placed STHTCs with dimensions of 30 mm × 30 mm × 2 mm on the CPU surface, resulting in a reduction in CPU temperature from 76.2 °C to 61.1 °C (Figure 18d,e), a reduction of 15.1 K. At the same time, stretchable thermoelectric flowing gel thermal cells can output voltages up to ~43.5mV, which confirms the potential of cooling devices and power generation applications, providing the opportunity to conduct thermal management simultaneously.

### 5.6. Integration with Other Systems

**Figure 19:**
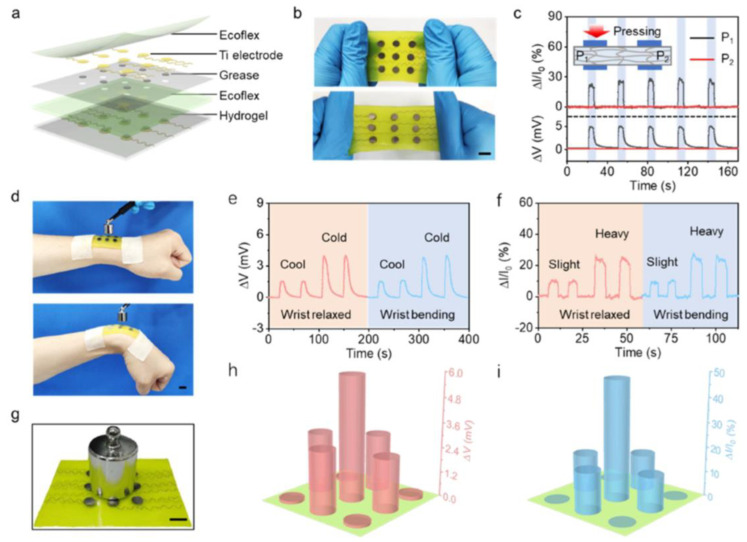
Design and performance of the TGH sensor array. (**a**) Schematic of a 3 × 3 pixel TGH sensor array. (**b**) Tensile properties of the TGH sensor array. (**c**) Response of output thermal voltage and relative current change of adjacent pixels on a TGH sensor array when one of them is pressed by a finger. (**d**) Photographs of the TGH sensor array as a secondary i-skin on the human wrist with relaxed and bending (strain: ∼25%) states. Output voltage (**e**) and relative current change (**f**) response curves generated by objects with different temperature and pressure. (**g**) Schematic of simultaneous measurement of temperature and pressure stimuli when a weight is placed in the center of a TGH sensor array. Output voltage (**h**) and relative current change (**i**) of the TGH sensor array in (**g**). All scale bars represent 1 cm. (Reproduced with permission from [124]. Copyright 2022 American Chemical Society).

Current research shows that thermogalvanic hydrogel can not only collect heat independently but can also be integrated with other systems to achieve synergistic enhancements. Fu et al. proposed a scalable self-powered temperature–pressure dual sensing skin based on thermogalvanic hydrogel (TGHs) [124], as shown in Figure 19. TGHs are obtained by introducing the redox coupling agent K_4_[Fe(CN)_6_]/[K_3_Fe(CN)_6_] into polyacrylamide hydrogels and, by combining the thermo–current and piezoresistive effects of TGHs, temperature, and pressure stimuli, can be converted into voltage and current signals, thus enabling the simultaneous monitoring of these two indicators.

## 6. Opportunities, Challenges, and Future Directions

In the past hundred years, human society has been plagued by the energy crisis and environmental problems. However, the energy shortage will become even more severe, and the environment will become worse in the future [125,126,127,128]. Due to the limitation of technology, a large amount of energy is wasted in the form of waste heat [129,130]. Thermogalvanic hydrogels, due to their thermal–electrical conversion capability, have been widely studied in recent years [131,132,133,134,135,136]. In a thermogalvanic hydrogel, the temperature dependence of the redox pair results in a massive amount of thermal energy capable of providing the conditions for continuous energy conversion. Moreover, optimizing the coupling of enhanced entropy values with electrodes can greatly improve their performance [137]. Additionally, it is possible to increase the thermoelectric energy by optimizing the way they contact with the environment. The functional design of thermogalvanic hydrogels makes them more suitable for future heat harvesting and wearable self-powered electronic devices. Combining the advantages, disadvantages, and current situation, some ideas are presented below for the further development of thermogalvanic hydrogels.

### 6.1. Opportunities

In the past few years, thermogalvanic hydrogels have made significant advances in both thermoelectric conversion and thermoelectric sensing [138,139,140,141]. Compared with other energy devices, such as chemical batteries, supercapacitors, and solar cells, first of all, thermogalvanic hydrogels can directly convert heat into electricity, enabling the recovery and utilization of applied waste heat, thus contributing to the development of green energy. In addition, many forms of heat energy sources are abundant and inexhaustible, such as solar energy, geothermal heat, industrial waste heat, and human body heat. There is no need to worry about the consumption of fossil fuels and the emission of toxic gases. Secondly, the utilization of water in thermogalvanic hydrogels provides the advantage of low cost and mass production, providing a convenient way for commercialization. More importantly, by using different gel electrolytes, transparent, flexible, stretchable, recyclable, and tough thermogalvanic hydrogels can be fabricated, which shows excellent potential in wearable and self-powered electronics. Thus, thermogalvanic hydrogels will be one of the most promising green energy applications, including waste heat harvesting, power generation, wearable and self-powered electronics, self-powered heat sensing, etc.

### 6.2. Challenges

Although thermogalvanic hydrogels show great potential for thermoelectric conversion and other applications, various challenges remain. While using water gives thermogalvanic hydrogels an advantage, it also has a detrimental effect on their practical application. One of the biggest challenges is that the water in water-based thermogalvanic hydrogel freezes at low temperatures or evaporates violently at high temperatures, resulting in a narrow operating temperature for thermogalvanic hydrogels. Therefore, it is essential to broaden the operating temperature of the thermoelectric conversion. Although the addition of antifreeze (e.g., LiCl and propanetriol) can expand the operating temperature, we especially hope it will expand to high temperatures.

In addition, lower energy conversion efficiency and thermoelectric figures of merit are among the challenges of thermogalvanic hydrogels. Therefore, great efforts are still needed to explore promising technologies. In addition to improving the Seebeck coefficient (Se) by the strategies mentioned above, efforts should be made to improve the conductivity (σ) because σ, in gel electrolytes, is only at the mS cm^−1^ level. This seriously hinders the performance of thermogalvanic hydrogels. Researchers should improve σ without sacrificing Se and k. Therefore, effective strategies can be investigated from electrode optimization since σ responds to the kinetics of chemical reactions. In terms of k suppression, other strategies should be proposed not only by creating thermal barriers and using low thermal conductivity electrolytes but also for promising gel electrolytes. In addition, discovering new redox pairs with good performance is also a good strategy.

The output thermogalvanic hydrogels have achieved is relatively long and stable production compared to semiconductor thermocouples, but it is still a challenge to achieve sound power output for days on end. Therefore, the packaging of the gel device has become one of the key factors affecting the performance of thermogalvanic hydrogel. It makes sense to use better packaging materials to broaden their applications beyond just charging capacitors and sensing heat signals for short periods of time.

### 6.3. Future Directions

Considering the advantages as well as the disadvantages of thermogalvanic hydrogels, the potential applications of thermogalvanics in the field of temperature detection and cooling deserve further investigation. In terms of temperature detection, hydrogel-based thermogalvanics have broad prospects as flexible temperature passive sensors in the fields of electronic skin, human body sign monitoring, human motion, and human-machine interface.

## Figures and Tables

**Table 1:** Comparison of the Seebeck coefficient, electrical conductivity, power density, and number of charge–discharge cycles.

Redox Couple	Matrix	Se (mV K^−1^)	σ (S m^−1^)	Pmax/(ΔT)^2^ (mW m^−2^K^−2^)	Number of Charging–Discharging Cycles	Ref.
Fe^3+^/Fe^2+^	PVA/Gelatin	1.63	0.75	0.03	30	[21]
Fe^3+^/Fe^2+^	HCl/PVA	1.02	1	0.01	/	[56]
[Fe(CN)_6_]^3−^/[Fe(CN)_6_]^4−^	KCl/Gelatin	17	1	0.66	50	[23]
[Fe(CN)_6_]^3^/[Fe(CN)_6_]^4−^	PVA	1.21	0.6	0.04	/	[56]
I^3−^/I^−^	Cyclodextrins/aqueous	1.9	2.4	/	/	[75]
I^3−^/I^−^	PVA/Gelation	0.63	0.06	/	5	[45]

## Data Availability

No new data were created or analyzed in this study. Data sharing is not applicable to this article.
